# Risk Factors and Prevalence Associated With Conversion of Laparoscopic Cholecystectomy to Open Cholecystectomy: A Tertiary Care Hospital Experience in Western Mexico

**DOI:** 10.7759/cureus.45720

**Published:** 2023-09-21

**Authors:** Lourdes I Ochoa-Ortiz, Enrique Cervantes-Pérez, Sol Ramírez-Ochoa, Alejandro Gonzalez-Ojeda, Clotilde Fuentes-Orozco, Itze Aguirre-Olmedo, Liliana F De la Cerda-Trujillo, Fernando M Rodríguez-Navarro, Eliseo Navarro-Muñiz, Gabino Cervantes-Guevara

**Affiliations:** 1 Department of Surgery, Hospital Civil de Guadalajara Juan I. Menchaca, Guadalajara, MEX; 2 Department of Internal Medicine, Hospital Civil de Guadalajara Fray Antonio Alcalde, Guadalajara, MEX; 3 Department of Clinics, Centro Universitario de Tlajomulco, Universidad de Guadalajara, Tlajomulco de Zuñiga, MEX; 4 Biomedical Research Unit 02, Specialties Hospital - Western National Medical Center, Mexican Institute of Social Security, Guadalajara, MEX; 5 Department of Health-Illness, Universidad de Guadalajara, Tlajomulco de Zuñiga, MEX; 6 Department of Surgery, Hospital México Americano, Guadalajara, MEX; 7 Department of Welfare and Sustainable Development, Centro Universitario del Norte, Universidad de Guadalajara, Guadalajara, MEX; 8 Department of Gastroenterology, Hospital Civil de Guadalajara Fray Antonio Alcalde, Guadalajara, MEX

**Keywords:** laparoscopic surgery, acute cholecystitis, risk factors, surgery, laparoscopic cholecystectomy

## Abstract

Introduction

Laparoscopic cholecystectomy (LC) is a common procedure used for the treatment of different pathologies caused by gallstones in the gallbladder, and one of the most common indications is acute cholecystitis. The definitive treatment for acute cholecystitis is surgery, and LC is the gold standard. Nevertheless, transoperative complications (like intraoperative bleeding, anatomical abnormalities of the gallbladder, etc.) of LC and some other preoperative factors (like dilatation of bile duct, increased gallbladder wall thickness, etc.) can cause or be a risk factor for conversion to open cholecystectomy (OC). The objective of this study was to determine the risk factors and prevalence associated with the conversion from LC to OC in patients with gallbladder pathology and the indication for LC.

Materials and methods

This was a prospective cohort study. We included patients of both sexes over 18 years of age with gallbladder disease. To determine the risk factors associated with conversion, we performed a bivariate analysis and then a multivariate analysis.

Results

The rate of conversion to OC was 4.54%. The preoperative factors associated with conversion, in the bivariate analysis, were common bile duct dilatation (p=0.008), emergency surgery (p=0.014), and smoking (p=0.001); the associated intraoperative variables were: laparoscopic surgery duration (p <0.0001), Calot triangle edema (p=0.033), incapacity to hold the gallbladder with atraumatic laparoscopic tweezers (p=0.036), and choledocholithiasis (p=0.042). Laparoscopic Surgery duration was the only factor with a significant association in the multivariate analysis (p=0.0036); we performed a receiver operating characteristic (ROC) curve analysis and found a cut-off point of 120 minutes for the duration of laparoscopic surgery with a sensitivity and a specificity of 67 and 88%, respectively.

Conclusion

The prevalence of conversion from LC to OC is similar to that reported in the international literature. The risk factors associated with conversion to OC, in this study, should be confirmed in future clinical studies, in this same population, with a larger sample size.

## Introduction

In Mexico, the incidence and prevalence of biliary lithiasis are not exactly known; however, autopsy studies suggest an approximate prevalence of 14.3% (20.4% in women and 8.5% in men, with a possible significant increase in the coming years) [1]. In the United States, it is estimated that 10-15% of the adult population suffers from cholelithiasis and that approximately 80,000 new cases are diagnosed every year [2]. In Latin America, it is reported that between 5 and 15% of the population has vesicular lithiasis, and countries such as the United States, Chile, and Bolivia are among the countries with the largest number of people affected by this disease [3].

Laparoscopic cholecystectomy (LC) is a common procedure used for the treatment of different pathologies caused by gallstones in the gallbladder, and one of the most common indications is acute cholecystitis [4,5]. LC is considered to have many economic, aesthetic, and functional advantages over open cholecystectomy (OC) in its results and recovery time [6]; nevertheless, transoperative complications of LC and technical (those that refer to the surgical procedure itself), patient (demographic data or medical history), or surgeon factors (surgeon experience) can cause the conversion to OC [7].

The frequency of conversion from LC to OC has remained constant, with a reported incidence ranging from 1% to 10%; nevertheless, in developing countries, there have been reports of conversion rates up to 22% [8,9]. OC is primarily selected to treat complicated cases of gallbladder disease for which LC fails or is contraindicated. Even when some relative contraindications have been reported, such as a history of previous abdominal surgery, Mirizzi syndrome (type 2), and terminal liver disease, among others, it is considered that there is little evidence to take them into account in the first instance since the benefit of laparoscopic intervention is greater than the risk that could arise; therefore, currently, only the diagnosis of invasive gallbladder carcinoma, uncorrected coagulopathy, and inability to tolerate general anesthesia or laparotomy are considered as absolute contraindications [10]. It is relevant to mention that delaying the decision to convert from LC to OC is associated with higher mortality [1,11].

The conversion rates have been reported in several articles worldwide [12-16], and, in several studies, researchers have tried to identify factors that predict the risk of conversion from LC to OC [13,17,18]. Although there are many factors associated with LC conversion to OC, there is no consensus on factors with any real clinical significance [8].

It is important to identify these predictors and causes in a timely manner to prevent patient complications during the procedure and unnecessary cost increases. For this, we chose to use the 10-point intraoperative gallbladder scoring system (G10) as a reference in the evaluation and prediction of conversion from LC to OC [19], however, not all the parameters evaluated in the complete score could be used (this is better described in the discussion section), so it only served as a guide regarding some important risk factors to evaluate in our patients. This score reflects the severity of cholecystitis by emphasizing four parameters: the appearance of the gallbladder (distended or contracted), ease of access, presence of sepsis in the peritoneal cavity (pus or bile), and cholecystoenteric fistula [19]. One of the reasons for choosing this score as a guide, in terms of the risk factors for conversion to OC to be evaluated, is that despite the existence of other proposed scores, which are even validated, where the risk prediction focuses on clinical and preoperative findings, the score proposed by Sugrue et al. highlights the importance of intraoperative factors [2].

The primary objective of this study was to determine the risk factors associated with conversion from LC to OC in patients with gallbladder disease and the rate of conversion in a population of Mexican patients, with the goal of having a better understanding of the rates of conversion in our population of patients and to highlight some points of possible intervention in the future for better planning of low-risk LC.

## Materials and methods

The study was approved by the Ethics Committee of the Hospital Civil de Guadalajara “Dr. Juan I. Menchaca” (protocol code R-230/21, August 09, 2021), and informed consent was obtained from each patient before undergoing procedures associated with the study. The study was conducted in accordance with the Declaration of Helsinki.

We prospectively enrolled patients admitted to the unit of General Surgery of the Hospital Civil de Guadalajara “Dr. Juan I. Menchaca” (HCG) between September and December 2021, and all patients were diagnosed with gallbladder pathology and planned to undergo LC.

Patients needed to meet all the following inclusion criteria to enter the study: patients of either sex who were over 18 years of age, provided informed consent, had a diagnosis of gallbladder disease (cholelithiasis, acute lithiasic cholecystitis, acute acalculous cholecystitis, gallbladder dyskinesia and/or Mirizzi syndrome), requested laparoscopic cholecystectomy to treat their gallbladder disease and underwent ultrasound examination of the liver, gallbladder, and bile ducts by a radiologist before the surgical procedure. Patients with one or more of the following exclusion criteria were excluded: patients with a presurgical diagnosis of untreated choledocholithiasis who needed intervention one or more weeks prior to inclusion in this study, and patients with a diagnosis of bile duct cancer and/or bile duct fistulas (it diagnosed by ultrasonography due to not having available the possibility of doing it by contrast-enhanced computed tomography). Patient demographics, clinical, laboratory, and ultrasonographic data, and outcomes were extracted from the medical records.

Despite being a high-volume unit, our surgical department does not have access to some methods or useful laboratory studies that are typically used for diagnosing biliary pathology, especially those required prior to or during cholecystectomy, such as magnetic resonance retrograde cholangiopancreatography (MRCP) or intraoperative cholangiogram. In the event that they are recommended for a particular case, the patient can possibly pay for them by hiring an external provider to carry out the study (this expense is not covered by public health insurance); however, most of the patients who present to our hospital have limited economic resources.

During surgery, all the intraoperative findings were recorded, and whether there was conversion to OC was registered. Additionally, we analyzed the association of some intraoperative factors that Sugrue et al. [2] previously described to be strongly associated with difficult LC and, hence, may be able to predict conversion to OC.

When pus or bile was detected outside the gallbladder or there were intraoperative findings compatible with an infectious process, 1 g of intravenous cefazolin was administered, followed by the same dose at 6 and 12 hours after anesthesia induction for the surgical process, as a mitigation measure according to the internal standardized procedures of our hospital unit.

Statistical analysis

For the data processing and descriptive and inferential statistical analysis, we used GraphPad Prism (version 9.3.1.471, GraphPad by Dotmatics, America). The inferential analysis for the qualitative variables was performed with the Chi-square test to compare the proportions of categorical dichotomous variables.

The normality and distribution of the quantitative variables of interest were established with the Kolmogorov‒Smirnov test using a parametric analysis. For the comparison variables between the patients with and without conversion to OC, we used the Student’s t-test. All p values <0.05 were considered statistically significant.

Multivariate analysis was carried out using an unconditional logistic regression model expressed as an odds ratio (OR). To test the independence of the risk factors, the variables considered significant in the bivariate analysis (P < 0.05) were entered into a multivariate logistic regression model with likelihood ratio backward selection and a significance criterion of P < 0.05. To determine the ability of the only significant independent variable to predict the dependent variable, a receiver operating characteristic (ROC) curve was generated.

The sample size was calculated using the formula for infinite populations, considering a confidence interval of 95% (CI 95%), with an estimated LC conversion rate of 4.5%, in accordance with the data reported in different studies with similar populations [12], and an error margin of 5%. This resulted in a total (n) of 66 patients being needed.

## Results

We included 66 patients in this study. The average age of the patients was 38.87 ± 15.08 years old (18 to 76 years old). The mean surgical time was 69.85 ± 6.65 minutes, with a range from 30 minutes to 300 minutes. The rate of conversion from LC to OC in this study was 4.54% (n=3).

To determine the preoperative and intraoperative factors associated with conversion, we performed a bivariate analysis (the results can be found in Table 1) and then a multivariate analysis (the results can be found in Table 2).

**Table 1:** Comparison of preoperative, surgical, and intraoperative characteristics between patients with and without conversion to open surgery SD = standard deviation; BMI = body mass index; OR = odds ratio; CI = confidence interval; mm = millimeters

Preoperative characteristics				
Characteristic	Conversion, n=3 (SD)	No Conversion, n=63 (SD)	OR (CI 95%)	p-value
Age (years)	32.33 ± 12.74	39.19 ± 15.20	-	0.445
BMI (kg/m2)	26.53 ± 8.40	28.12 ± 5.65	-	0.642
Gallbladder wall thickness (mm)	4.73 ± 2.19	3.47 ± 1.09	-	0.066
Common bile duct dilatation (mm)	8.93 ± 7.95	4.35 ± 2.53	-	0.008
Male gender, %	33.33	20.63	0.52 (0.05-8.06)	0.599
Acute cholecystitis, %	66.67	23.81	6.40 (0.68-94.19)	0.097
History of abdominal surgery, %	0.0	41.27	-	0.152
Emergency surgery, %	33.33	3.17	15.25 (0.71-163)	0.014
History of pancreatitis, %	0.0	6.35	-	0.6525
History of choledocholithiasis, %	33.33	6.35	7.37 (0.41-70.19)	0.084
Comorbidities, %	0.0	28.57	-	0.277
Smoking, %	33.33	1.59	31 (1.10-588)	0.001
Surgical characteristics				
Characteristic	Conversion, n=3 (SD)	No Conversion, n=63 (SD)	OR (CI 95%)	p value
Laparoscopic surgery duration (minutes)	200 ± 91.65	57.90 ± 18.17	-	<0.0001
Third-year general surgery resident, %	66.67	85.71	0.33 (0.03-5.32)	0.368
Intraoperative characteristics				
Characteristic	Conversion (n=3)	No Conversion (n=63)	OR (CI 95%)	p value
Calot triangle edema, %	100.0	38.10	-	0.033
Pyocholecystitis, %	0.0	14.29	-	0.481
Anatomical malformation of the gallbladder, %	33.33	7.94	5.80 (0.33-54.21)	0.134
Adherences, %	66.67	50.79	1.93 (0.21-28.87)	0.590
Choledocholithiasis, %	33.33	4.76	10 (0.52-98.44)	0.042
Gallbladder neck stone impaction, %	33.33	14.29	3 (0.18-27.27)	0.368
Bile or pus outside the gallbladder, %	33.33	25.40	1.46 (0.09-13.20)	0.758
Inability to grab the gallbladder with atraumatic laparoscopic tweezers/the need for gallbladder decompression to hold it with grasper, %	66.67	17.46	9.45 (0.98-138.70)	0.036

**Table 2:** Associated factors for conversion to open surgery, multivariate analysis (binary logistic regression) OR = odds ratio; CI = confidence interval

Factor	OR (CI 95%)	p-value
Common bile duct dilatation	1.10 (0.71-1.55)	0.616
Emergency surgery	0.59 (0.40-0.72)	0.999
Smoking	0.50 (0.40-0.60)	0.880
Laparoscopic surgery duration	1.013 (1.009-1.02)	0.036
Calot triangle edema	0.68 (0.58-0.77)	0.303
Choledocholithiasis (intraoperative diagnosis)	2.61 (0.01-298.1)	0.717
Inability to grab the gallbladder with atraumatic laparoscopic tweezers/the need for gallbladder decompression to hold it with a grasper	0.57 (0.47-0.67)	0.133

No mitigation measures were reported during the intraoperative process prior to the decision to convert to OC. Of the total sample, 17 patients had acute cholecystitis (25.65%); of these, three patients underwent urgent surgery (the rest were treated with antibiotics prior to surgery, which was performed within the first 72 days of the diagnosis of acute cholecystitis), of which only one patient required conversion to OC.

**Figure 1:**
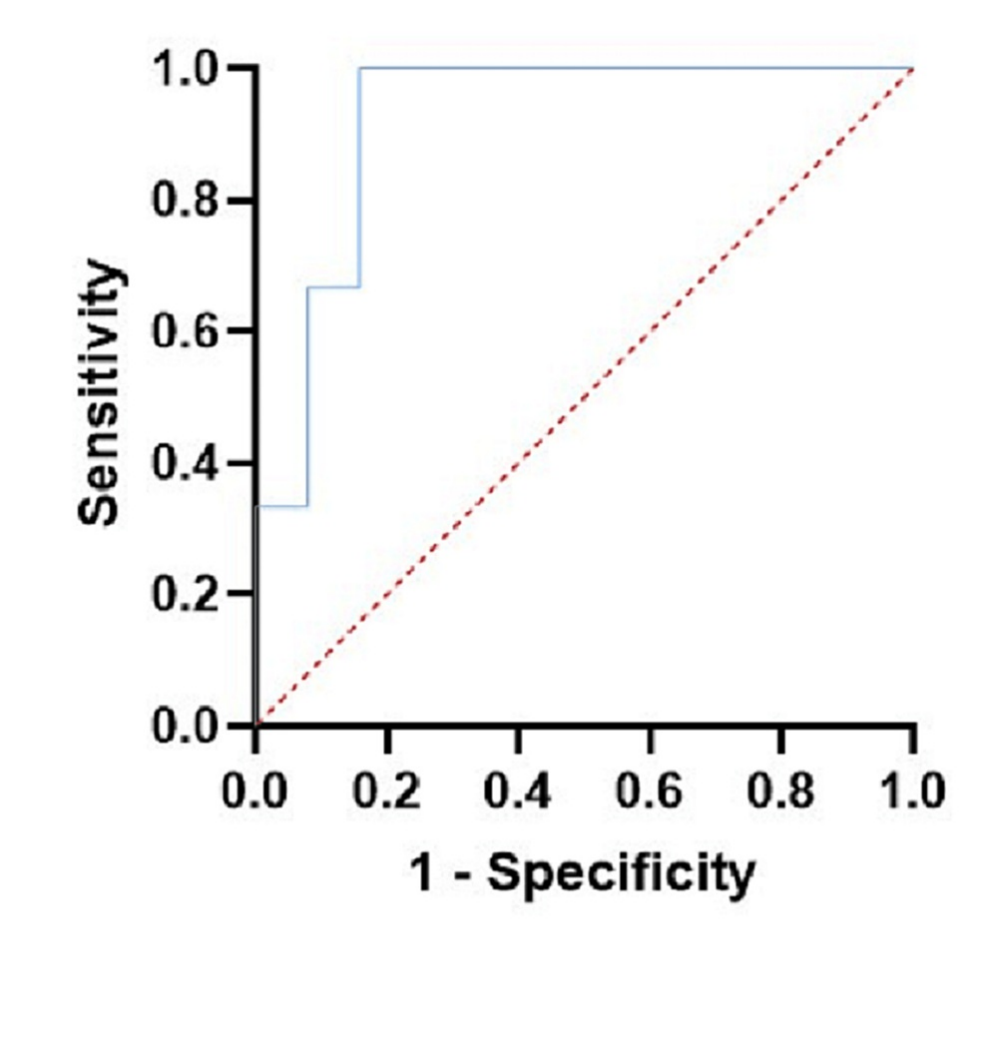
ROC curve for prediction of conversion from LC to open surgery using the surgery duration ROC: receiver operating characteristic; LC: laparoscopic cholecystectomy

A multivariate analysis was carried out to determine the independent factors associated with conversion from LC to OC through binary logistic regression with a backward step model of entry and the Hosmer‒Lemeshow goodness-of-fit test [20]. There was only one variable that was independently associated with conversion to OC, namely, the duration of laparoscopic surgery; therefore, we generated a receiver operating characteristic (ROC) curve to determine the prediction capacity. We found an area under the curve (AUC) of 0.920 (p=0.014; Figure 1). The best cutoff point was a duration of 120 minutes, in which the sensitivity was 100% and the specificity was 84.1%.

## Discussion

The rate of conversion to OC was 4.54%, which was in accordance with that reported elsewhere (national and international) [12-16]. It was lower than the rate reported in some Australian and North American studies [21,22], but it was slightly higher than that reported in some other Mexican hospitals (2.7-3.8%) [17]. In a 2015 meta-analysis of 10 clinical studies conducted from 1993 to 2012, the average conversion rate was 20.87% [23]; likewise, it can be seen that, over time, the rate decreased, which may be due to the techniques used and the accumulation of surgical experience over the years, which could explain the higher rate of conversion in the first year [23]. In our study, there was no significant difference between groups regarding the years of experience (surgical training) of the surgeon who performed the procedure. All the patients who were included were operated on by physicians in their last year of training in the general surgery specialty (which in Mexico would correspond to the third year of training) or by general surgeons assigned to the general surgery service with at least three years of subsequent experience since their training in the specialty.

The preoperative factors associated with conversion to OC in the bivariate analysis were bile duct dilatation (diameter), emergency surgery, and previous smoking. These findings are similar to those reported by Jansen et al. [24], who found that dilatation of the bile duct > 6 mm and gallbladder wall thickness > 4 mm were predictors for conversion from LC to OC. In this study, we did not find a significant association with gallbladder wall thickness.

Other preoperative factors that have typically been associated with conversion from LC to OC in other studies [1,8,19,24,25] were not significantly associated with conversion in this study (Table 1). One of the intraoperative findings commonly related to conversion to OC is the presence of intraoperative bleeding (defined as blood loss exceeding 1000 mL or requiring a blood transfusion) or intraoperative hemorrhage of the bed of the gallbladder [26]. None of our patients included in this study presented bleeding, and thus, it was not possible to associate it with the risk of conversion.

In the study by Thesbjerg et al. [27], it was observed that men had a 2.48 times higher risk of conversion from laparoscopic to OC, with a frequency of 13.3% compared to an incidence of 5.8% in females; however, in our study, there was no significant difference. Of all the intraoperative variables analyzed in the bivariate analysis, only laparoscopic surgery duration, Calot triangle edema, incapacity to hold the gallbladder with atraumatic laparoscopic tweezers/necessity for decompression of the gallbladder, and choledocholithiasis showed a significant association with conversion from LC to OC. This is in contrast to other factors reported elsewhere, such as adherence syndrome, gallbladder inflammation, empyema, anatomical abnormalities of the gallbladder, and gallbladder distention [28].

The mitigation processes prior to the conversion to OC, in the subjects who required conversion, were not reported during the intraoperative process; even though we can assume that this was due to the absence of these measures, it is necessary to specify whether it was not a component of the data asked promptly at the time of postsurgical data collection. In future studies, it could be a variable of interest to include in the risk analysis.

In the medical literature, we could not find an actual association between the duration of laparoscopic surgery and the conversion from laparoscopic to OC. In the previous publication by Sugrue et al., the category of cystic artery and duct identification time (with a cutoff point of > 90 minutes) was taken into account, which was replaced by limited access due to adhesions from previous surgeries [19]. In our study, the duration of laparoscopic surgery turned out to have a marked association in the multivariate analysis; therefore, we performed a ROC curve analysis and found a regular AUC for the prediction cutoff, with a sensitivity and specificity of 100 and 84.1%, respectively, for a surgery time greater than 120 minutes. This result must be interpreted with caution, since, despite being significantly associated in our two analyses (both bivariate and multivariate), the increase in risk is very low and the sample of patients is very small. In addition, this variable is not a primary association risk factor, it is rather a variable that reflects that, as the difficulty of surgery increases, either due to preoperative factors of the patient or intraoperative factors of both the patient and the procedure, the surgical time lengthens (secondary to these factors either individually or together) and this, in turn, indicates that there is a greater possibility of conversion to OC, so that the duration of laparoscopic surgery is truly a variable dependent on several factors, which they might be a more adequate explanation in themselves of the conversion to OC. A larger sample could give us greater certainty as to whether any associated factor or perhaps the combination of certain specific factors increases the difficulty of surgery, the duration of the procedure, and consequently, the risk of conversion to OC.

In a study of 5,164 consecutive cases of laparoscopic cholecystectomies, Genc et al. [26] evaluated the causes of conversion from LC to OC and found that inflammatory adhesions were the main cause, followed by fibrosis of the Calot triangle and intraoperative hemorrhage of the bed of the gallbladder. Other less common causes were the migration of stones to the peritoneal space or the thickening of the wall of the gallbladder [26]. The rare causes included the presence of a cholecystoduodenal fistula (0.08%), bleeding from cystic artery lesions (0.04%) or hepatic lesions (0.02%), damage to the bile ducts (0.02-0.06%), duodenal or colonic drilling (0.02%), stones in the common bile duct (0.02%), and suspicion of malignancy (0.02%) [26].

LC has obvious advantages over OC (shorter hospital stay, quicker recuperation, less pain, better cosmetic results, etc.) [1], and it is important to identify the risk factors that can lead to OC conversion. For this study, we took into special consideration the intraoperative characteristics used in the G10 score proposed by Sugrue et al. [2,19], unfortunately, due to the lack of documentation of some of the characteristics evaluated in this score, we could not calculate it as such in the vast majority of patients, so this, coupled to the small size of our sample, resulted in an insufficient number of patients for which the calculation could be made. This deficiency in our study can be used to highlight the importance of documenting surgical findings in order to make a better analysis of outcomes [2], and that, at least in our sociodemographic context, it is not a commonly performed practice. The G10 score was used as a reference for evaluating the factors that can predict conversion of the surgical procedure since it solidly includes real intraoperative findings, can be easily implemented in clinical settings and is relevant and simple to use, thereby broadening its use [19]. We found some associations among the characteristics that could be evaluated and the risk of conversion to OC in this study. In the future, we can use this complete scoring scale, which, coincidentally, has been recently validated [29], to determine the severity of cholecystitis that indicates the degree of difficulty for LC and then evaluate its association, since this is already a complex variable that takes into account several intraoperative risk factors, to the risk of conversion to OC.

Among the strengths of our study, it can be highlighted that it has a prospective design, which usually has fewer potential sources of bias and confounding than retrospective studies, which are the most frequently reported studies concerning the risk of conversion from LC to OC [1-9,15,18,24,25,30].

Among the limitations of the study, it should be noted that due to the limited access to and the costs associated with MRCP, intraoperative cholangiogram, and contrast-enhanced computed tomography, our patient population did not have access to these procedures, which could have affected some of the outcomes in the study. Additionally, even though the sample size was adequate in terms of the objective of this study, the associations that were found could be better elucidated by improved statistical robustness that only comes from a larger sample size; unfortunately, small samples do not contain enough information to make reliable inferences about the shape of the distribution of the population from which they originate, therefore, the differences between groups that we found in our study must be interpreted with caution, and it must be specially considered that the sample of patients who converted to OC is very small, and although the results shown can give us an idea of risk factors, that may require further attention, only a study with a larger sample size could provide us with more reliable associations. Another limitation of our study was that, unfortunately, we were unable to perform the recently validated G10 score calculation [2,19,29], as mentioned previously, due to the lack of recorded interoperative information on the patients included in our study.

It is possible that other factors associated with conversion from LC to OC can be found with a larger sample size [30]. However, this is the first reported rate of conversion in our hospital (with population from western Mexico), and we consider that this is a relevant area of study that could have an impact not only on the recuperation time but also on the financial burden of patients who undergo LC, especially in developing countries, where local health statistics for different illnesses and medical complications are often unknown.

## Conclusions

The rate of conversion to OC, in our study, was 4.54%. Several risk factors were found to have a significant association with conversion to OC, both preoperative and intraoperative, which are worth confirming in future studies and with a larger sample size.

In the multivariate analysis, the duration of laparoscopic surgery showed a significant association with the conversion of the surgical procedure, a result that could influence the consideration of including it among the predictive factors of conversion from laparoscopic to OC, especially when no other specific factors can be identified, to maintain some readiness during the surgical procedure. More clinical studies are needed, with a larger sample size, to have a greater statistical certainty of the true factors associated with the conversion to OC, but studies like ours can provide a good starting point that highlights the importance of investigating this issue in greater depth.
